# *Dendrobium chrysotoxum* Lindl. Alleviates Diabetic Retinopathy by Preventing Retinal Inflammation and Tight Junction Protein Decrease

**DOI:** 10.1155/2015/518317

**Published:** 2015-01-01

**Authors:** Zengyang Yu, Chenyuan Gong, Bin Lu, Li Yang, Yuchen Sheng, Lili Ji, Zhengtao Wang

**Affiliations:** ^1^The Shanghai Key Laboratory of Complex Prescription and The MOE Key Laboratory for Standardization of Chinese Medicines, Institute of Chinese Materia Medica, Shanghai University of Traditional Chinese Medicine, Shanghai 201203, China; ^2^Center for Drug Safety Evaluation and Research, Shanghai University of Traditional Chinese Medicine, Shanghai 201203, China

## Abstract

Diabetic retinopathy (DR) is a serious complication of diabetes mellitus. This study aimed to observe the alleviation of the ethanol extract of *Dendrobium chrysotoxum* Lindl. (DC), a traditional Chinese herbal medicine, on DR and its engaged mechanism. After DC (30 or 300 mg/kg) was orally administrated, the breakdown of blood retinal barrier (BRB) in streptozotocin- (STZ-) induced diabetic rats was attenuated by DC. Decreased retinal mRNA expression of tight junction proteins (including occludin and claudin-1) in diabetic rats was also reversed by DC. Western blot analysis and retinal immunofluorescence staining results further confirmed that DC reversed the decreased expression of occludin and claudin-1 proteins in diabetic rats. DC reduced the increased retinal mRNA expressions of intercellular adhesion molecule-1 (ICAM-1), tumor necrosis factor *α* (TNF*α*), interleukin- (IL-) 6, and IL-1*β* in diabetic rats. In addition, DC alleviated the increased 1 and phosphorylated p65, I*κ*B, and I*κ*B kinase (IKK) in diabetic rats. DC also reduced the increased serum levels of TNF*α*, interferon-*γ* (IFN-*γ*), IL-6, IL-1*β*, IL-8, IL-12, IL-2, IL-3, and IL-10 in diabetic rats. Therefore, DC can alleviate DR by inhibiting retinal inflammation and preventing the decrease of tight junction proteins, such as occludin and claudin-1.

## 1. Introduction

Diabetic retinopathy (DR) is one of the most common and serious complications of diabetes mellitus (DM). With the improvement of people's living standards, the incidence of diabetes has increased in developed and developing countries, including China [1]. Patients who suffer from diabetes for a long time likely experience chronic vascular injury caused by high blood sugar levels; these patients are also at risk of DR when chronic vascular injury occurs in the retina [2, 3]. The pathogenesis of DR is generally divided into two stages, namely, nonproliferative DR (NPDR) and proliferative DR (PDR). In early NPDR stage, DR is characterized by retinal blood vessel permeability as a result of damaged blood retinal barrier (BRB); in PDR stage, retinal neoangiogenesis occurs [2, 3]. Previous studies have demonstrated that both inflammation and angiogenesis play important roles in regulating the development of DR, and thus anti-inflammation and antiangiogenesis are potential therapeutic strategies for the treatment of DR [4, 5]. It is worth mentioning that, in the opinion of Adamis, DR is considered as an inflammatory disease [6].

*Dendrobium* is one of the largest genera in the family of Orchidaceae [7]. More than 70 species of* Dendrobium* are found in the mountain ranges of southern and western China; some of these species are reported to exhibit antioxidant, immunomodulatory, anticancer, and antisenescence activities [8]. In traditional Chinese medicine, Shi-Hu is a famous and precious Chinese herbal medicine derived from different species of* Dendrobium*, including* D. chrysotoxum* Lindl.,* D. nobile* Lindl., and* D. fimbriatum* Hook.. Shi-Hu has been used to prepare various medicinal and health products in many Asian countries.* D. chrysotoxum* is a commonly used species of medicinal* Dendrobium* (Shi-Hu), indexed in the Chinese Pharmacopoeia (2010 version). There is report found that polysaccharides from* D. chrysotoxum* had antihyperglycemic and antioxidant effects [9]. Furthermore, a series of aromatic compounds, such as bibenzyls and fluorenones, were reported to be isolated from* D. chrysotoxum* [10, 11]. Among those compounds, bibenzyl erianin is reported to exhibit antiangiogenic activity and has the potential capacity to be developed into anticancer drug [12, 13].

In our previous study, the ethanol extract isolated from* D. chrysotoxum* (DC) alleviates retinal angiogenesis during the development of DR [14]. In the present study, we aimed to observe whether DC can ameliorate streptozotocin- (STZ-) induced DR in rats by alleviating retinal inflammation and restoring the decreased expression of tight junction proteins.

## 2. Materials and Methods

### 2.1. Chemicals and Reagents

*D. chrysotoxum* was a gift from Jinling Pharmaceutical Co., LTD (Nanjing, China). The ethanol extract of* D. chrysotoxum* (DC) was prepared, as described in our previous study [14].

Enzyme-linked immunosorbent assay (ELISA) kits were purchased from RapidBio (West Hills, CA). Trizol reagent was purchased from Life Technology (Carlsbad, CA). PrimeScript RT Master Mix and SYBR Premix Ex Taq were purchased from Takara (Shiga, Japan). Intercellular adhesion molecule-1 (ICAM-1) antibody was purchased from Biobasic Inc. (Shanghai, China). Phospho-p65, phospho-I*κ*B, and phospho-I*κ*B kinase (IKK) antibodies were purchased from Cell Signaling Technology (Danvers, MA). The chemiluminescent kit for western blot analysis was purchased from Millipore (Billerica, MA). Occludin and claudin-1 antibodies were purchased from Santa Cruz (Santa Cruz, CA). 4′, 6-Diamidino-2-phenylindole (DAPI), Alexa Fluor 488 goat anti-mouse IgG (H+L) antibody and Cy3 goat anti-rabbit IgG (H+L) antibody were purchased from Life Technology (Carlsbad, CA). Pierce BCA Protein Assay Kit was purchased from ThermoFisher Scientific (Waltham, MA). All of the other reagents were purchased from Sigma (St. Louis, MO), unless otherwise is indicated.

### 2.2. Experimental Animals

Sprague-Dawley rats (160–200 g) were purchased from Shanghai Laboratory Animal Center of Chinese Academy of Sciences (Shanghai, China). These rats were maintained under controlled temperature (23 ± 2°C), humidity (50%), and lighting (12 h light/12 h dark) conditions. The rats were fed with a standard laboratory diet and given free access to tap water. All animals received humane care according to the Institutional Animal Care guidelines approved by the Experimental Animal Ethical Committee of Shanghai University of Traditional Chinese Medicine.

### 2.3. Animals Treatment

A total of 65 rats were randomly divided into two groups. Among these rats, 50 rats were administered intraperitoneally (i.p.) with 65 mg/kg STZ, and other 15 rats were i.p. injected with physiological saline; and the latter served as control rats. Serum glucose concentration was measured after 7 d. Rats with high glucose concentration (>16.5 mmoL/L) were considered as diabetic rats. In this experiment, the glucose concentration of 44 rats was >16.5 mmoL/L; these rats were randomly divided into three groups: DR model (*n* = 14), DC (30 mg/kg) (*n* = 15), and DC (300 mg/kg) (*n* = 15). Two months after STZ was injected, the rats were orally treated with DC (30 or 300 mg/kg per day) for one month. The rats in the control group and the DR model group were orally administered with vehicle control. Three months after STZ was injected, six rats from each group were selected to evaluate BRB breakdown by using Evans blue dye. The other rats were anesthetized with sodium pentobarbital (40 mg/kg, i.p.). Blood samples were collected from the abdominal aorta, and the eyes were removed immediately. Body weight and serum glucose concentration were monitored during the whole experiment.

### 2.4. Evaluation of BRB Breakdown by Using Evans Blue Dye

BRB breakdown was evaluated according to previously reported method [15] with some modifications. In brief, the rats were injected with 2% Evans blue dye (10 *μ*l/g, i.p.) in PBS. 2 h later, blood was extracted through the left ventricle and the rats were perfused with PBS to completely remove Evans blue dye from blood vessels. The retinas were carefully dissected and thoroughly dried; afterward, the weight of the retinas was measured. The retinas were then incubated in 120 *μ*l formamide for 18 h at 70°C to extract Evans blue dye. The extract was centrifuged twice at 10,000 ×g for 1 h at 4°C. The absorbance of the extract was determined using a spectrophotometer at 620 nm. The concentration of Evans blue dye in the extracts was calculated using a standard curve of Evans blue dye in formamide and then normalized to the dried retinal weight.

### 2.5. RNA Isolation and cDNA Synthesis

Retinas were carefully dissected from the eyeball by using forceps. Total RNA of the retina was isolated using Trizol reagent according to the manufacturer's instructions. cDNA was synthesized according to the manufacturer's instructions described in PrimeScript RT Master Mix kits.

### 2.6. Real-Time PCR Analysis

The mRNA expressions of occludin (Ocln), ZO-1 (Tjp1), claudin-1 (Cldn1), claudin-5 (Cldn5), ICAM-1 (ICAM1), TNF*α* (Tnf), IL-6 (Il6), IL-1*β* (Il1b), and actin (Actb) were quantified by real-time PCR. Real-time PCR was performed using StepOne Plus (Carlsbad, CA) with SYBR green premix in accordance with the manufacturer's instructions. Relative target gene expressions were normalized to Actb (actin), analyzed by 2^−ΔΔCt^ method, and expressed as ratio relative to the control. The primer sequences used in this study are shown in Table 1.

### 2.7. Immunofluorescence Staining of Occludin and Claudin-1

Paraffin-embedded retinal sections (5 *μ*m) were deparaffinized in xylene and rehydrated in an ethanol gradient with distilled water. After endogenous peroxidase activity was quenched, retinal sections were incubated with 5% bovine serum albumin to minimize nonspecific binding. Retinal sections were incubated with occludin or claudin-1 antibody at 4°C overnight and were further incubated with Cy3 goat anti-rabbit IgG (H+L) antibody or Alexa Fluor 488 goat anti-mouse IgG (H+L) antibody at room temperature for 1 h. The nuclei were stained with DAPI; images were captured under an inverted microscope (Nikon, Japan).

### 2.8. Western Blot Analysis

Retinas (proximately 20 mg) were homogenized in ice-cold lysis buffer containing 50 mM Tris (pH7.5), 1 mM EDTA, 150 mM NaCl, 20 mM NaF, 0.5% NP-40, 10% glycerol, 1 mM phenylmethylsulfonyl fluoride, 10 *μ*g/ml aprotinin, 10 *μ*g/ml leupeptin, and 10 *μ*g/ml pepstatin A. Homogenates were centrifuged at 3,000 ×g for 10 min before the supernatant was transferred to new tubes. Protein concentrations in the supernatants were assayed by BCA Protein Assay Kit and every sample was normalized to equal protein concentration. Proteins were separated by SDS-PAGE and transferred to immobilon-P PVDF membranes (Millipore). The membranes were blocked with 5% nonfat milk in TBST for 1 h and then were incubated with respective primary antibodies at 4°C overnight. The membranes were subsequently washed with TBST and incubated with horseradish peroxidase conjugated secondary antibodies for 1 h at room temperature; afterward, the membranes were visualized using a chemiluminescent kit.

### 2.9. ELISA Analysis

Whole blood was allowed to stand at room temperature for 2 h and then centrifuged at 3,000 rpm, at 4°C for 15 min. Serum was collected to detect the concentrations of TNF*α*, IL-6, IL-1*β*, IFN*γ*, IL-8, IL-12, IL-2, IL-3, and IL-10 by ELISA according to the manufacturer's instructions. Cytokines concentrations in the respective samples were determined on the basis of standard curves prepared using recombinant cytokines of known concentrations.

### 2.10. Statistical Analysis

Data were expressed as means ± standard deviation (SD). The significance of differences between groups was evaluated by one-way ANOVA with LSD post hoc test when the significance of the test of homogeneity of variances is above 0.05. Otherwise, the significance of differences between groups was evaluated by nonparametric tests with Mann-Whitney *U* test. *P* < 0.05 was considered as statistically significant difference.

## 3. Results

### 3.1. Measurement of Body Weight and Blood Glucose Concentration

**Figure 1:**
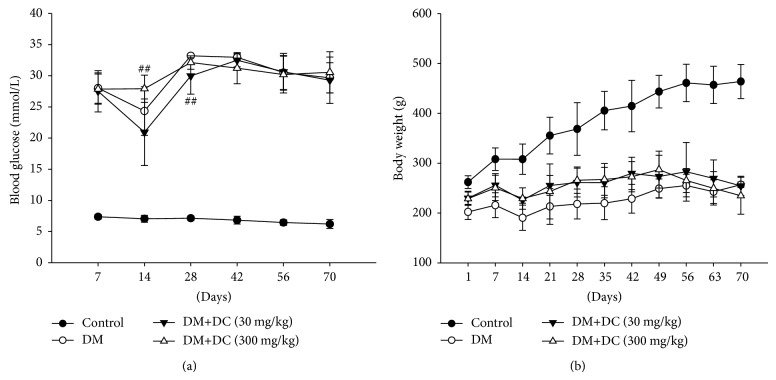
Analysis of serum glucose level and body weight. (a) Serum glucose level; (b) body weight. Data are expressed as means ± SD (*n* = 10 for control, *n* = 7 for DM, *n* = 9 for DM+DC 30 mg/kg, and *n* = 8 for DM+DC　300 mg/kg). ^##^ *P* < 0.01 compared to DM without DC.

Serum glucose concentrations in all of the groups were significantly different from the control rats during the experimental periods (Figure 1(a)). DC (30 or 300 mg/kg) was administrated two months after STZ was injected. As shown in Figure 1, we can see that DC (30 and 300 mg/kg) had no effect on the increased serum glucose concentration in STZ-induced diabetic rats. Furthermore, the body weights of STZ-induced diabetic rats were lower than those of the normal control rats (*P* < 0.001) (Figure 1(b)). No evident differences were observed between diabetic rats with or without the treatment of DC (30 and 300 mg/kg).

### 3.2. Evaluation of BRB Breakdown by Using Evans Blue Dye

**Figure 2:**
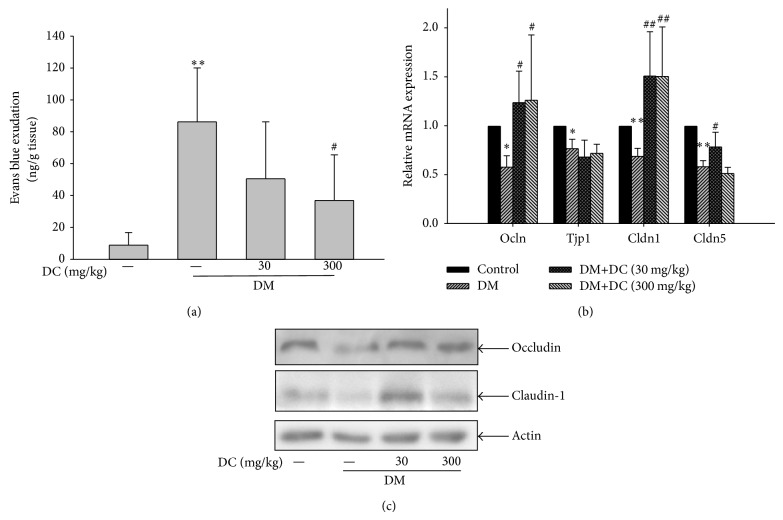
(a) Quantitative results of retinal blood vessel leakage by Evans blue dye. Data are expressed as means ± SD (*n* = 6). ^***^ *P* < 0.001 compared to control; ^###^ *P* < 0.001 compared to DM without DC. (b) Retinal mRNA expression of occludin (Ocln), ZO-1 (Tjp1), claudin-1 (Cldn1), and claudin-5 (Cldn5). Data are expressed as means ± SD (*n* = 10 for control, *n* = 7 for DM, *n* = 9 for DM+DC 30 mg/kg, and *n* = 8 for DM+DC 300 mg/kg). ^*^ *P* < 0.05, ^**^ *P* < 0.01 compared to control; ^#^ *P* < 0.05, ^##^ *P* < 0.01 compared to DM without DC. (c) Retinal protein expression of occludin and claudin-1 is detected by immunoblotting using specific antibodies. The occludin protein represents four independent experiments, and claudin-1 protein represents two independent experiments.

Increased leakages of Evans blue dye were observed in the retinas of STZ-induced diabetic rats (*P* < 0.01) (Figure 2(a)). This result indicates the increased retinal vessel leakage. After the rats were treated with 300 mg/kg DC, the increased retinal vessel leakage was reduced (*P* < 0.05). However, 30 mg/kg DC did not inhibit the increased retinal vessel leakage in STZ-induced diabetic rats.

### 3.3. Retinal mRNA and Protein Expressions of Tight Junction Proteins

The mRNA expressions of tight junction proteins were further analyzed by real-time PCR. The retinal mRNA expressions of occludin (Olcn), ZO-1 (Tjp1), claudin-1 (Cldn1), and claudin-5 (Cldn5) were decreased in STZ-induced diabetic rats (*P* < 0.05, *P* < 0.05, *P* < 0.01, and *P* < 0.01, resp.). DC (30 and 300 mg/kg) reversed the decreased retinal mRNA expression of occludin in STZ-induced diabetic rats (*P* < 0.05) (Figure 2(b)); by contrast, DC did not evidently affect the decreased ZO-1 expression. The decreased retinal mRNA expression of claudin-1 in STZ-induced diabetic rats was increased after these rats were treated with DC (30 and 300 mg/kg) (*P* < 0.01) (Figure 2(b)). However, the decreased mRNA expression of claudin-5 in the STZ-induced diabetic rats remained unchanged after the rats were treated with 300 mg/kg DC, but 30 mg/kg DC could weakly increase the decreased expression of claudin-5 (*P* < 0.05). The protein expression of occludin and claudin-1 was decreased in the STZ-induced diabetic rats; conversely, DC (30 and 300 mg/kg) reversed the decreased protein expressions of occludin and claudin-1 (Figure 2(c)).

### 3.4. Retinal Immunofluorescence Staining of Occludin and Claudin-1

**Figure 3:**
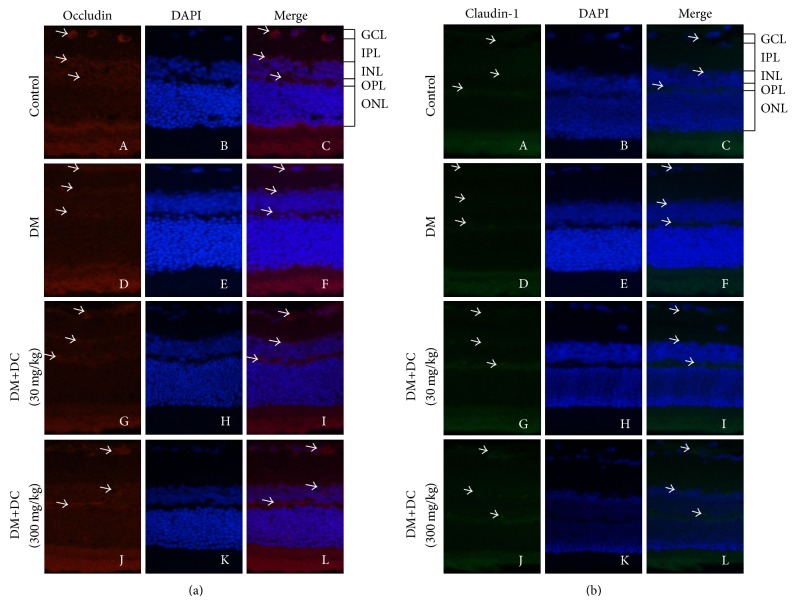
The expression of occludin and claudin-1 in retinas. (a) The representative pictures of retinal immunofluorescence staining of occludin (A, D, G, and J) and DAPI (B, E, H, and K). Merge of occludin- and DAPI-stained images (C, F, I, and L). (b) The representative pictures of retinal immunofluorescence staining of claudin-1 (A, D, G, and J) and DAPI (B, E, H, and K). Merge of claudin-1- and DAPI-stained images (C, F, I, and L) (original magnification ×400).

Occludin and claudin-1 were subjected to immunofluorescence staining to detect their corresponding expressions in the retinas. DAPI staining results (Figures 3(a) and 3(b)-B, E, H, and K) showed ganglion cell layer (GCL), inner plexiform layer (IPL), inner nuclear layer (INL), outer plexiform layer (OPL), and outer nuclear layer (ONL) in the retinas. The retinas stained with occludin were also observed (Figure 3(a)-A, D, G, and J). After the occludin-stained images were merged with DAPI-stained images, we can see that the retinal expression of occludin was decreased in GCL, INL, and OPL (Figure 3(a)-F); this decrease was reversed by DC (30 and 300 mg/kg) (Figure 3(a)-I and L). The retinas stained with claudin-1 were also observed (Figure 3(b)-A, D, G, and J). After the claudin-1-stained images were merged with DAPI-stained images, we can see that the retinal expression of claudin-1 was decreased in GCL, INL, and OPL (Figure 3(b)-F); this decrease was reversed by DC (30 and 300 mg/kg) (Figure 3(b)-I and L).

### 3.5. Retinal mRNA Expressions of ICAM-1, TNF-*α*, IL-6, and IL-1*β*

**Figure 4:**
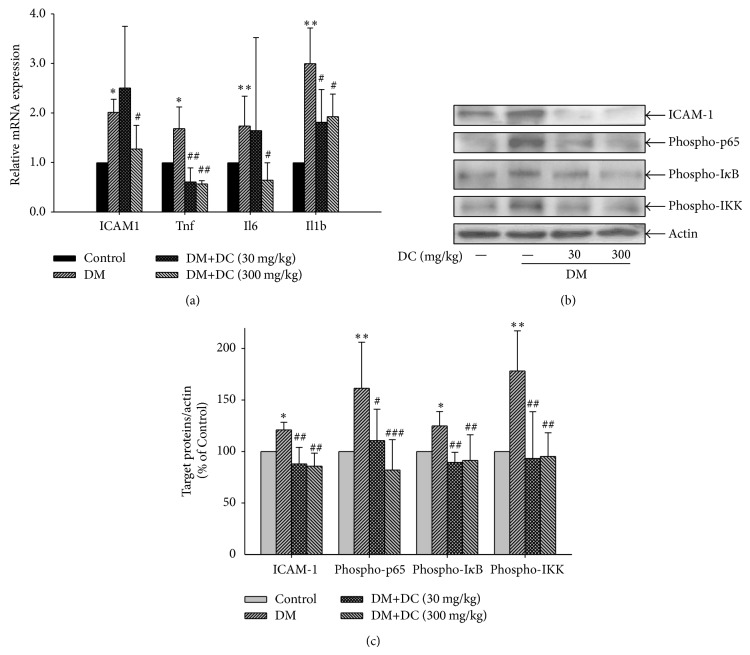
Effects of DC on the expression of ICAM-1, TNF*α*, IL-6, IL-1*β*, and the activation of NF-*κ*B signaling pathway. (a) Retinal mRNA expression of ICAM-1 (ICAM1), TNF*α* (Tnf), IL-6 (Il6), IL-1*β* (Il1b). Data are expressed as means ± SD (*n* = 10 for control, *n* = 7 for DM, *n* = 9 for DM+DC 30 mg/kg, and *n* = 8 for DM+DC 300 mg/kg). ^*^ *P* < 0.05, ^**^ *P* < 0.01 compared to control; ^#^ *P* < 0.05, ^##^ *P* < 0.01 compared to DM without DC. (b) Retinal expression of ICAM-1 and phosphorylated p65, I*κ*B, and IKK. ICAM-1 and phosphorylated p65, I*κ*B, and IKK are detected by immunoblotting using specific antibody. Results represent at least three repeated experiments. (c) Quantitative densitometric analysis of ICAM-1 and phosphorylated p65, I*κ*B, and IKK. Data are expressed as means ± SD (*n* = 10 for control, *n* = 7 for DM, *n* = 9 for DM+DC 30 mg/kg, and *n* = 8 for DM+DC 300 mg/kg). ^*^ *P* < 0.05, ^**^ *P* < 0.01 compared to control; ^#^ *P* < 0.05, ^##^ *P* < 0.01, and ^###^ *P* < 0.001 compared to DM without DC.

The retinal mRNA expressions of ICAM-1 (ICAM1), TNF*α* (Tnf), IL-6 (Il6), and IL-1*β* (Il-1b) were increased in the STZ-induced diabetic rats (*P* < 0.05, *P* < 0.01) (Figure 4(a)). DC (300 mg/kg) reduced the increased retinal mRNA expressions of ICAM-1, TNF*α*, IL-6, and IL-1*β* (*P* < 0.05, *P* < 0.01). DC (30 mg/kg) also reduced the increased retinal mRNA expressions of TNF*α* and IL-1*β* (*P* < 0.05, *P* < 0.01).

### 3.6. Retinal Expression of ICAM-1 and Phosphorylation of p65, I*κ*B, and IKK

The protein expression of ICAM-1 was increased in the retinas of STZ-induced diabetic rats, but it was decreased in DC-treated (30 and 300 mg/kg) diabetic rats (*P* < 0.01) (Figures 4(b) and 4(c)). In addition, the expressions of the phosphorylated p65, I*κ*B, and IKK were increased in the STZ-induced diabetic rats (*P* < 0.05, *P* < 0.01), but this increase was decreased after the rats were treated with DC (30 and 300 mg/kg) (*P* < 0.05, *P* < 0.01, *P* < 0.001, resp.).

### 3.7. Serum Cytokine Levels

**Figure 5:**
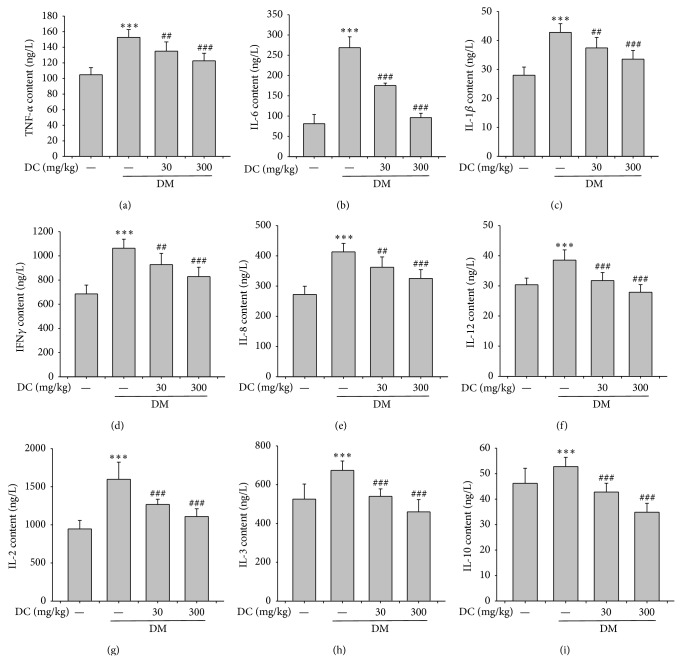
Serum levels of various cytokines. (a) TNF*α*, (b) IL-6, (c) IL-1*β*, (d) IFN*γ*, (e) IL-8, (f) IL-12, (g) IL-2, (h) IL-3, and (i) IL-10. Data are expressed as means ± SD (*n* = 10 for control, *n* = 7 for DM, *n* = 9 for DM+DC 30 mg/kg, and *n* = 8 for DM+DC 300 mg/kg). ^***^ *P* < 0.001 compared to control; ^##^ *P* < 0.01, ^#^ *P* < 0.05 compared to DM without DC.

The ELISA results showed that the serum levels of TNF*α*, IFN-*γ*, IL-6, IL-1*β*, IL-8, IL-12, IL-2, IL-3, and IL-10 were increased in the STZ-induced diabetic rats (*P* < 0.001) (Figure 5). DC (30 and 300 mg/kg) reduced the increased serum levels of those cytokines (*P* < 0.01, *P* < 0.001); and the inhibitory effect of 300 mg/kg DC was better than that of 30 mg/kg DC.

## 4. Discussion

According to the historical records of traditional Chinese medicine, Shi-Hu can be used to improve eyesight. In general, Shi-Hu is a monarch drug of several Chinese patent drugs, such as Shi-Hu-Ye-Guang-Wan and Shi-Hu-Ming-Mu-Wan, which are traditionally used to improve eyesight and indexed in Chinese and Local Pharmacopeia. Although Shi-Hu is traditionally used to improve eyesight in China, there are no much scientific evidences supporting its activity and mechansim. One report demonstrated the amelioration of total alkaloids extracted from* D. nobile* on diabetic cataract in rats [16]. Recently, our previous study showed that* D. chrysotoxum* alleviated retinal angiogenesis during the development of DR [14]. Our results in this study showed that DC did not evidently affect body weight and blood glucose concentration of diabetic rats; however, 300 mg/kg DC alleviated retinal BRB breakdown in STZ-induced diabetic rats. The results provided additional evidences regarding the potential amelioration of* D. chrysotoxum* on DR; these results also contributed to the treatment of DR affecting diabetic patients.

ZO-1, occludin, and claudin-1/5 are the main proteins involved in the tight junction complex, which is essential for the control of endothelial permeability [17, 18]. Retinal endothelial barrier integrity is also implicated in the maintenance of vascular permeability and the development of DR [19]. ZO-1, occludin, and claudin-1/5 expressions are decreased in NPDR [20, 21]. Our results showed the decreased retinal mRNA expression of ZO-1, occludin, and claudin-1/5 in diabetic rats, but the decreased expressions of occludin and claudin-1 were reversed by DC (30 and 300 mg/kg). Furthermore, the reversed protein expressions of occludin and claudin-1 induced by DC were confirmed by western blot analysis and immunofluorescence staining results of occludin and claudin-1 in the retinas. These results indicated that DC could prevent the decreased expression of tight junction proteins, such as occludin and claudin-1, which may also contribute to the alleviation of DC on STZ-induced NPDR.

ICAM-1 is critical for regulating retinal leukocyte adhesion, and its expression is induced by NF-*κ*B activation mediated by proinflammatory cytokines; this process has been correlated with the increase in leukostasis and further breakdown of BRB in the development of DR [22]. Our results demonstrated that DC (300 mg/kg) reduced the increased retinal mRNA expression of ICAM-1. Furthermore, DC (30 and 300 mg/kg) reduced the increased protein expression of ICAM-1 in STZ-induced diabetic rats. Therefore, DC could prevent retinal vascular leakage in the development of STZ-induced DR.

Multiple mediators play important roles in inflammation, including proinflammatory cytokines and chemokines. Inflammation is critically involved in the development of DR [12, 13]. TNF*α*, IL-6, and IL-1*β* are common proinflammatory cytokines; these cytokines are increased in serum, vitreous, or retinas from diabetic patients or rats [23–25]. This increase in proinflammatory cytokine concentration in diabetic rats can be decreased by* Panax notoginseng*,* Radix Astragali*, and* Angelica sinensis* aqueous extracts,* Tinospora cordifolia* extract, and compounds, including *α*-linolenic acid and curcumin, and these substances elicit obviously beneficial effects by preventing the development of DR [26–29]. Furthermore, NF-*κ*B/Rel family plays a critical role in regulating host immune and inflammatory responses by regulating the production of various pro-inflammatory cytokines, such as TNF*α* and IL-1*β*, and so forth [30, 31]. Our results showed that DC reduced the increased retinal mRNA expression and serum levels of TNF*α*, IL-6, and IL-1*β*, and also reduced the increased phosphorylation of p65, I*κ*B, and IKK in STZ-induced diabetic rats. Therefore, DC ameliorated retinal inflammation by inhibiting NF-*κ*B signaling pathway.

Other cytokines, such as IL-8, IFN-*γ*, IL-2, and IL-3, are increased in patients or rats with DR [32–35]. In the present study, DC (30 and 300 mg/kg) prevented the increased serum levels of these cytokines, which may also contribute to the amelioration of DC on DR. IL-10 is considered as an anti-inflammatory cytokine; however, serum or intravitreal IL-10 levels are not remarkably different in diabetic patients and nondiabetic patients [35–37]. Conversely, decreased IL-10 level has been observed in diabetic patients [38]. IL-12 was previously reported to inhibit angiogenesis via inducing IP-10 expression [39]. No difference in serum IL-12 level has been observed in nondiabetic patients and diabetic patients [40]; however, decreased serum IL-12 level has been detected in some diabetic patients [41]. Despite these contradictory results, sera IL-10 and IL-12 levels have not been detected in STZ-induced diabetic rats. Our results showed that sera IL-10 and IL-12 concentrations were increased in STZ-induced diabetic rats but decreased in DC-treated diabetic rats. The increased sera IL-10 and IL-12 levels may be attributed to the enhanced self-defense of diabetic rats during the development of DR to counteract inflammation and angiogenesis. As DR was ameliorated by DC, the increased IL-10 and IL-12 levels were also alleviated.

**Figure 6:**
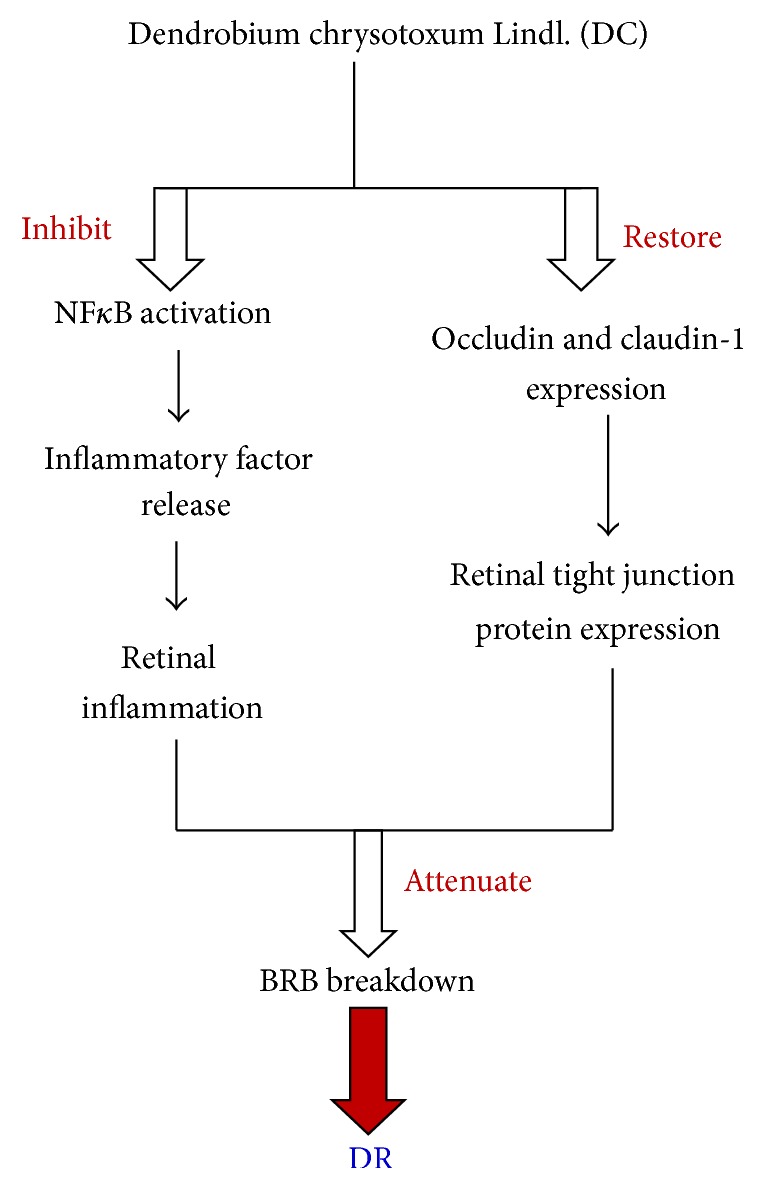
The schematic diagram of mechanism involved in the amelioration of DC against DR in STZ-induced diabetic rats.

Our results demonstrated that DC inhibited retinal inflammation by inhibiting NF-*κ*B signaling pathway. DC also prevented the decreased expression of tight junction proteins, such as occludin and claudin-1. These findings greatly contributed to the amelioration of DC against the development of DR. The schematic diagram of mechanism involved in the amelioration of DC against DR was shown in Figure 6. The present study and previous study [14] demonstrate that* D. chrysotoxum* could ameliorate retinal inflammation, maintain retinal BRB, and attenuate retinal angiogenesis, thereby attenuating the development of DR. Thus,* D. chrysotoxum* may be recommended as a supplementary treatment for patients with DR. These results also provided strong experimental evidences for the eyesight-improving capacity of medicinal* Dendrobium* (Shi-Hu).

## Figures and Tables

**Table 1:** List of primers for real-time PCR.

Target	Gene ID	Primer	Sequence
ICAM1	25464	FP	5′-TGCAGGTGAACTGCTCTTCCTCTT-3′
		RP	5′-AGCTTCCAGTTGTGTCCACTCGAT-3′

Tnf	24835	FP	5′-CCACCACGCTCTTCTGTCTACTG-3′
		RP	5′-GGGCTACGGGCTTGTCACTC-3′

Il6	24498	FP	5′-TCCTACCCCAACTTCCAATGCTC-3′
		RP	5′-TTGGATGGTTCTTGGTCCAATGCTC-3′

Il1b	24494	FP	5′-TGTCACTCATTGTGGCTGTGGAGA-3′
		RP	5′-TGGGAACATCACACACTAGCAGGT-3′

Ocln	83497	FP	5′-TGGGACAGAGCCTATGGAACG-3′
		RP	5′-ATCACCAAGGAAGCGATGAAGC-3′

Tjp1	292994	FP	5′-GATGAGCGGGCTACCTTATTGA-3′
		RP	5′-AGCGAACTGAATGGTCTGATGC-3′

Cldn1	65129	FP	5′-TGCCCTACTTTCCTGCTCCTGT-3′
		RP	5′-AGTAGAAGGTGTTGGCTTGGGATAA-3′

Cldn5	65131	FP	5′-CAGCGTTGGAAATTCTGGGTCT-3′
		RP	5′-CGTCTGCGCCGTCACGATAT-3′

Actb	81822	FP	5′-CGCTCGTCGTCGACAACGG-3′
		RP	5′-TGTGGTGCCAAATCTTCTCC-3′

FP: forward primer; RP: reverse primer.
